# Supplementary material for: A Critical View of the Application of the APEX Software (Aqueous Photochemistry of Environmentally-Occurring Xenobiotics) to Predict Photoreaction Kinetics in Surface Freshwaters
Source: Molecules. 2019 Dec 18;25(1):9. doi: 10.3390/molecules25010009 (PMC7017383; doi:10.3390/molecules25010009)
Supplement: Supplementary file 1 [file molecules-25-00009-s001.zip › 3.2.4_gcc-4.4.0.zip!/3.2.4_gcc-4.4.0/3.2.4_gcc-4.4.0/doc/octave/html/interpreter/Overloading-and-Autoloading.html]

Overloading and Autoloading - Untitled


Next: Function Locking,
Previous: Private Functions,
Up: Function Files


---

#### 11.7.4 Overloading and Autoloading

The `dispatch` function can be used to alias one function name to
another. It can be used to alias all calls to a particular function name
to another function, or the alias can be limited to only a particular
variable type. Consider the example

```
     function y = spsin (x)
       printf ("Calling spsin\n");
       fflush(stdout);
       y = spfun ("sin", x);
     endfunction
     
     dispatch ("sin", "spsin", "sparse matrix");
     y0 = sin(eye(3));
     y1 = sin(speye(3));
```

which aliases the user-defined function `spsin` to `sin`, but only for real sparse
matrices. Note that the builtin `sin` already correctly treats
sparse matrices and so this example is only illustrative.

— Loadable Function: **dispatch** (f, r, type)  
> Replace the function f with a dispatch so that function r
> is called when f is called with the first argument of the named
> type. If the type is any then call r if no other type
> matches. The original function f is accessible using
> `builtin (`f`, ...)`.
>
> If r is omitted, clear dispatch function associated with type.
>
> If both r and type are omitted, list dispatch functions
> for f.
>
>
>
> **See also:** builtin.

— Loadable Function: [...] **builtin** (f, ...)  
> Call the base function f even if f is overloaded to
> some other function for the given type signature.
>
>
>
> **See also:** dispatch.

A single dynamically linked file might define several
functions. However, as Octave searches for functions based on the
functions filename, Octave needs a manner in which to find each of the
functions in the dynamically linked file. On operating systems that
support symbolic links, it is possible to create a symbolic link to the
original file for each of the functions which it contains.

However, there is at least one well known operating system that doesn't
support symbolic links. Making copies of the original file for each of
the functions is undesirable as it increases the
amount of disk space used by Octave. Instead Octave supplies the
`autoload` function, that permits the user to define in which
file a certain function will be found.

— Built-in Function: **autoload** (function, file)  
> Define function to autoload from file.
>
> The second argument, file, should be an absolute file name or
> a file name in the same directory as the function or script from which
> the autoload command was run. file should not depend on the
> Octave load path.
>
> Normally, calls to `autoload` appear in PKG\_ADD script files that
> are evaluated when a directory is added to the Octave's load path. To
> avoid having to hardcode directory names in file, if file
> is in the same directory as the PKG\_ADD script then
>
> ```
>           autoload ("foo", "bar.oct");
> ```
>
> will load the function `foo` from the file `bar.oct`. The above
> when `bar.oct` is not in the same directory or uses like
>
> ```
>           autoload ("foo", file_in_loadpath ("bar.oct"))
> ```
>
> are strongly discouraged, as their behavior might be unpredictable.
>
> With no arguments, return a structure containing the current autoload map.
>
>
>
> **See also:** PKG\_ADD.
